# Correlation of Redox Status with Procalcitonin and C-reactive Protein in Septic Patients

**DOI:** 10.1155/2020/5147364

**Published:** 2020-09-04

**Authors:** Jasna Petrovic, Tamara Nikolic Turnic, Vladimir Zivkovic, Marijana Andjic, Nevena Draginic, Aleksandra Stojanovic, Ivan Milinkovic, Sergey Bolevich, Jasna Jevdjic, Vladimir Jakovljevic

**Affiliations:** ^1^General Hospital “Valjevo”, Valjevo, Serbia; ^2^University of Kragujevac, Faculty of Medical Sciences, Department of Pharmacy, Kragujevac, Serbia; ^3^University of Belgrade, Faculty of Medicine, Department of Cardiology, Clinical Center of Serbia, Serbia; ^4^I.M. Sechenoov First Moscow State Medical University, Department of Human Pathology, Moscow, Russia; ^5^University of Kragujevac, Faculty of Medical Sciences, Department of Anaesthesiology and Critical Care Medicine, Kragujevac, Serbia; ^6^University of Kragujevac, Faculty of Medical Sciences, Department of Physiology, Kragujevac, Serbia

## Abstract

Based on the role of oxidative stress in the pathophysiological mechanisms of sepsis and the importance of PCT as a clinically applicable biomarker for early detection of inflammatory response initiation, we aimed this study at examining the correlation between PCT levels and oxidative stress parameters (prooxidants and antioxidants) in patients with sepsis. This study was designed as a case-series prospective clinical study which involved 103 critically ill patients and 17 healthy participants with diagnosis of sepsis/septic shock (over 18 years of age, both gender) admitted to the Intensive Care Unit (ICU) of Valjevo General Hospital in Serbia. All subjects were divided into patients who were operated on/underwent surgery before sampling and have sepsis (*n* = 24), patients who were operated on/underwent surgery before sampling and have septic shock (*n* = 25), patients who were not operated on/did not undergo surgery before sampling and have sepsis (*n* = 26), patients who were not operated on/did not undergo surgery before sampling and have septic shock (*n* = 28), and participants who are healthy (*n* = 17). PCT has confirmed a positive correlation with prooxidants and type of critical illness, and performing surgical intervention diminished oxidative stress in patients with septic shock. Prognosis in critically ill patients was strongly associated with PCT levels but not with nonspecifically C-reactive protein.

## 1. Introduction

Sepsis is a global health problem. In addition to complex modern therapy, mortality in critically ill patients remains one of the leading causes [1]. At the Third International Consensus on the Definition of Sepsis and Septic Shock 2016, a consensus was established to define and treat sepsis (e.g., Sepsis-3). The new classification involves sepsis and septic shock. Sepsis has been defined as a life-threatening organic dysfunction caused by the body's uncontrolled response to infection [2–4]. Septic shock is a subtype of sepsis characterized by deepening circulatory, cellular, and metabolic dysfunction and carries a higher risk of fatal outcome than sepsis itself. Clinical characteristics of patients in septic shock are the inability to maintain mean arterial pressure (e.g., mean arterial blood pressure (MAP)) of >65 mmHg without a vasopressor under normovolemia conditions and serum lactate levels > 2 mmol/l [5].

During the development of sepsis, there is a disruption of the physiological functions of the interdependent organs, which can range from a mild degree of impaired function to complete, irreversible organ failure and is referred to as Multiple Organ Dysfunction Syndrome (MODS). The assessment of the severity of the organ dysfunction condition and treatment outcome is monitored via the SOFA score (e.g., SOFA (Sepsis-Related Organ Failure Assessment) score). Respiratory function, coagulation status, liver function, urinary system, state of consciousness, and hemodynamic parameters are evaluated. Systems score 1-4 (0 is a physiological state); the total score can be from 6 to 24 points. An increase of >2 parameters [4, 5] is considered positive.

Pathophysiological events during sepsis are complex. They aim to eliminate pathogens and restore the body's response to a state of homeostasis. Sepsis develops after an initial host response to an infection becomes amplified and dysregulated, which leads to circulatory changes and septic shock. The most common consequences are impaired vascular permeability, cardiac malfunction, and mitochondrial dysfunction leading to impaired respiration. The pathogenesis of sepsis-induced myocardial injury remains unclear, but the mitochondrial dysfunction of myocardial cells plays a very important role in the pathophysiological mechanism; oxidants and antioxidants also play a key role [5–8]. Normally, there is a balance between the oxidant and antioxidant systems in the body; oxidative stress occurs when oxidant levels exceed those of antioxidants, which contributes to the septic process and may lead to organ damage [5–8].

Then, in stimulated tissues, polymorphonuclear leukocytes infiltrate and activate the monocyte/macrophage system. This activation leads to increased production of reactive oxygen radicals (e.g., reactive oxygen species (ROS)) as well as reactive nitrogen radicals (e.g., reactive nitrogen species (RNS)) [9, 10]. Sepsis is a condition in which large amounts of nitric oxide (NO) are produced that have a direct inhibitory effect on the respiratory chain of mitochondria and their physical damage causing mitochondrial and endothelial dysfunction that exacerbates MODS [11, 12]. In addition, research suggests a role for delayed neutrophil apoptosis and prolonged neutrophil responses in the body's uncontrolled response to infection [13, 14].

Accordingly, earlier studies done in both patients and animals have shown that during sepsis, there are an increased concentration of prooxidants and a decrease in antioxidant protection. Patients with severe sepsis have also been confirmed to have higher levels of prooxidants than patients in the sepsis group [10].

Early diagnosis of sepsis and timely initiation of antibiotic therapy are significant for the outcome of the treatment of patients with sepsis and septic shock [15, 16]. Various biomarkers are currently used for the early confirmation of infection and tissue damage in sepsis: C-reactive protein, leukocyte level, lactate level, and procalcitonin (PCT). According to the recommendations of the 2012 sepsis treatment guide, procalcitonin has been proposed as one of the biomarkers that is useful as a guide to the inclusion of antibiotic therapy and monitoring of the effect and its duration. PCT concentration is detected after 2-4 hours from the onset of stroke. The highest values are in the first 6-24 hours and remain elevated for the next 7 days [17, 18]. Furthermore, sepsis is a lack of specific criteria of diagnosis in an early stage, and it is likely to develop to severe sepsis or septic shock. Patients with sepsis often undergo emergency surgery. For these septic patients, control of infection, involving removal of infected and necrotic tissue and surgical drainage of abscess combined with early antimicrobial therapy, is essential to the successful treatment of sepsis [19].

Nevertheless, there is little information available in the available literature on the correlation of procalcitonin and oxidative stress parameters. Based on the role of oxidative stress in the pathophysiological mechanisms of sepsis and the importance of PCT as a clinically applicable biomarker for the early detection of inflammatory response initiation, we aimed this study at examining the correlation between PCT levels and oxidative stress parameters (prooxidants and antioxidants) in patients with sepsis.

## 2. Patients and Methods

### 2.1. Ethical Concerns

The study was approved by the Local Ethics Committee at Valjevo General Hospital. All procedures were according to the Declaration of Helsinki (1964). Patients received written informed consent to participate in the study. In the event that the patient's general condition made it impossible to obtain consent, the consent was obtained from the closest relative.

### 2.2. Design of Study

This study was designed as a case-series prospective clinical study which involved 120 adult female and male participants (103 critically ill patients with diagnosis of sepsis/septic shock and 17 healthy participants) who were admitted to the Intensive Care Unit (ICU) of Valjevo General Hospital in Serbia. All patients are also divided according to the performed surgical intervention in terms of appropriate therapy for each of them, and all biochemical procedures (blood samples) are done after performing surgical intervention into the next groups:

1. Patients who were operated on/underwent surgery before sampling and have sepsis (*n* = 24)
2. Patients who were operated on/underwent surgery before sampling and have septic shock (*n* = 25)
3. Patients who were not operated on/did not undergo surgery before sampling and have sepsis (*n* = 26)
4. Patients who were not operated on/did not undergo surgery before sampling and have septic shock (*n* = 28)
5. Participants who are healthy (*n* = 17)

Patients under 18 years of age, pregnant women, patients undergoing immunosuppressive therapy, patients with transplanted organs, and patients in the terminal stage of malignancies would be excluded from the study.

### 2.3. Criteria for Sepsis Confirmation

Sepsis was defined as a change in SOFA score > 2. Septic shock is defined as sepsis requiring vasopressors to maintain MAP (mean arterial pressure) > 65 mmHg with serum lactate levels > 2 mmol/l [20]. The score (SOFA score) was calculated via an online calculator, http://ClinCalc.com, for Sequential Organ Failure Assessment (SOFA) [20]. Patients were included in the study when found to have sepsis on the day they met the Sepsis-3 criteria for sepsis (increase of ≥2 SOFA score). The first blood samples were taken that day. A single tube of blood has been taken from the patient in the same procedure and from the same vein pathway for analysis of oxidative stress parameters and determination of procalcitonin (PCT). Calculation of the SOFA score was repeated daily until blood sampling for PCT levels, and analysis of oxidative stress parameters was repeated within 24 hours of initial blood sampling and after 72 hours (3 time points were obtained). These time intervals were chosen due to the influence of confounding variables (initiation or termination of mechanical ventilation, subsequent introduction of a vasopressor, need for antibiotic change, need for surgical intervention, etc.).

### 2.4. Blood Sample Preparation

Immediately after collecting blood samples, serum, plasma, and hemolysate are separated from whole blood. For serum samples, blood was collected in a vacutainer, and after collection of the whole blood, the blood was allowed to clot by leaving it undisturbed for 30 minutes at room temperature. For plasma samples, we used commercially available anticoagulant-treated (citrate-treated) tubes (light blue tops). Clots or cells are removed from serum or plasma by centrifugation for 10 minutes at 1000–2000 × *g* using a refrigerated centrifuge. The samples are maintained at 2–8°C until handling. After plasma separation, red blood cells (RBCs) were then resuspended in saline and washed twice by centrifugation at 2000 rpm for 10 min. The washed RBCs were then resuspended in saline to the original volume of the blood sample.

### 2.5. Serum PCT Measurement

Serum PCT was measured by using the Enzyme-Linked Fluorescent Assay (ELFA) technique by using the VIDAS BRAHMS PCT test with range of detection of 0.05-200 ng/ml (BIOMERIEUX SA FRANCE, serial number ITV30101685). The used sample is a human serum (lithium heparinate), which was separated from the clot and stored at 2-8°C for 48 h until the determination. The Solid Phase Receptacle (SPR) container is in the form of a pipette and at the same time is used for pipetting and serves as an antibody carrier. The interior of the SPR was coated with mission monoclonal antihuman procalcitonin immunoglobulins. All test steps are performed automatically with the instrument itself. The reaction medium enters and leaves the SPR cyclically several times. The sample is pipetted into a cuvette containing an antiprocalcitonin antibody labeled with an alkaline phosphatase (conjugate). The sample/conjugate mixture cyclically enters and exits the SPR to increase the reaction rate. The antigen binds to both specific antibodies fixed to the surface of the SPR and to the conjugate forming a “sandwich.” Unbound components are eliminated during rinsing. The two phases of detection are performed successively. During each phase, the substrate (4-methyl-umbeliferyl phosphate) is cyclically introduced into and out of the SPR. The enzyme conjugate catalyzes the hydrolysis of this substrate to a fluorescent product (4-methyl-umbeliferon) whose fluorescence is measured at 450 nm. The intensity of fluorescence is proportional to the concentration of antigen present in the sample. PCT concentrations are expressed in ng per ml.

### 2.6. Determination of Prooxidative Markers (Superoxide Anion Radical (O_2_^−^), Hydrogen Peroxide (H_2_O_2_), Nitric Oxide (NO) by Determining Nitrate (NO_3_^−^) and Nitrite (NO_2_^−^), and TBARS)

The concentration of the superoxide anion radical (O_2_^−^) was measured after the reaction of nitro blue tetrazolium in Tris buffer with the plasma at 530 nm. Distilled water solution served as a blank. A concentration of superoxide dismutase is expressed in nmol/ml [21].

The measurement of hydrogen peroxide (H_2_O_2_) is based on the oxidation of phenol red by hydrogen peroxide, in a reaction catalyzed by horseradish peroxidase (HRPO). 200 *μ*l of plasma sample was precipitated with 800 ll of freshly prepared phenol red solution, followed by the addition of 10 *μ*l of (1 : 20) HRPO (made ex tempore). For the blank, distilled water, instead of the plasma samples, was used. The level of H_2_O_2_ was measured at 610 nm. Concentration of hydrogen peroxide is expressed in nmol/ml [22].

NO decomposes rapidly to form stable metabolite nitrite (NO^−^)/nitrate products. The method for detection of the plasma nitrate and NO^–^ levels is based on the Griess reaction. NO^–^ was determined as an index of NO production with the Griess reagent [23]. 0.1 ml 3 N perchloride acid, 0.4 ml 20 mmol/l ethylenediaminetetraacetic acid (EDTA), and 0.2 ml plasma were put on ice for 15 min and then centrifuged for 15 min at 6000 rpm. After pouring off the supernatant, 220 ll K CO was added. NO^–^ was measured at 550 nm. Distilled water was used as a blank probe. Concentration of nitrites is expressed in nmol/ml [23].

The degree of lipid peroxidation in the plasma samples was estimated by measuring TBARS using 1% thiobarbituric acid in 0.05 NaOH, incubated with the plasma samples at 100°C for 15 min, and measured at 530 nm. Distilled water solution with 1% thiobarbituric acid in 0.05 NaOH served as a blank probe. Concentration of TBARS is expressed in *μ*mol/ml [24].

### 2.7. Determination of Antioxidant Status

Isolated RBCs were washed 3 times with 3 vol. of ice-cold 0.9 mmol/l NaCl. Hemolysates containing about 50 g Hb/l (prepared according to Beutler [25, 26]) were used for the determination of SOD, GSH, and CAT activity. The haemoglobin content of each hemolysate was measured by the cyanmethemoglobin method.

Superoxide dismutase (SOD) activity was determined by the epinephrine method of Beutler. 100 *μ*l lysate and 1 ml carbonate buffer were mixed, and then, 100 *μ*l of epinephrine was added. Distilled water was used as a blank probe. Detection was performed at 470 nm. The SOD activity in the erythrocytes is expressed as units (U)/per gram of haemoglobin × 10^3^ [25].

Plasma level of reduced glutathione (GSH) was determined spectrophotometrically, and it is based on GSH oxidation via 5,5-dithiobis-6,2-nitrobenzoic acid. The GSH extract was obtained by combining 0.1 ml 0.1% EDTA, 400 ll plasma, and 750 ll precipitation solution containing 1.67 g metaphosphoric acid, 0.2 g EDTA, and 30 g NaCl and filled with distilled water until 100 ml. After mixing in the vortex machine and extraction on cold ice (15 min), it was centrifuged at 4000 rpm (10 min). Distilled water was used as a blank probe. Measuring was performed at 420 nm. The GSH activity in the erythrocytes is expressed as units (U)/per gram of haemoglobin × 10^3^ [26].

Catalase (CAT) activity was determined according to Aebi. Lysates were diluted with distilled water (1 : 7 *v*/*v*) and treated with chloroform ethanol (0.6 : 1 *v*/*v*) to remove haemoglobin. Then, 50 *μ*l CAT buffer, 100 *μ*l sample, and 1 ml 10 mM H_2_O_2_ were added to the samples. Distilled water was used as a blank probe. Detection was performed at 360 nm. The CAT activity in the erythrocytes is expressed as units (U)/per gram of haemoglobin × 10^3^ [27].

### 2.8. Statistical Analysis

Data are presented as mean values ± SEM. One-way ANOVA followed by the Tukey HSD test was used to analyse all data. A bivariate correlation as a statistical technique was used to determine the existence of relationships between two different variables. For all tests described above, a *p* < 0.05 was considered significant. SPSS (SPSS for Macintosh, version 25.0, USA) was used for data analysis.

## 3. Results

### 3.1. Demographic Characteristics of the Study Population

Distribution by gender and age according to the groups is presented in Table 1. In our study, males (60.02%) were predominant compared to females (39.98%). Also, the mean values of age in the study population were 67.53 ± 2.1 years. There were no statistically significant differences between distribution of gender and means of age (Table 1).

### 3.2. Oxidative Stress Marker Levels in Critically Ill Operated and Nonoperated Patients

**Figure 1:**
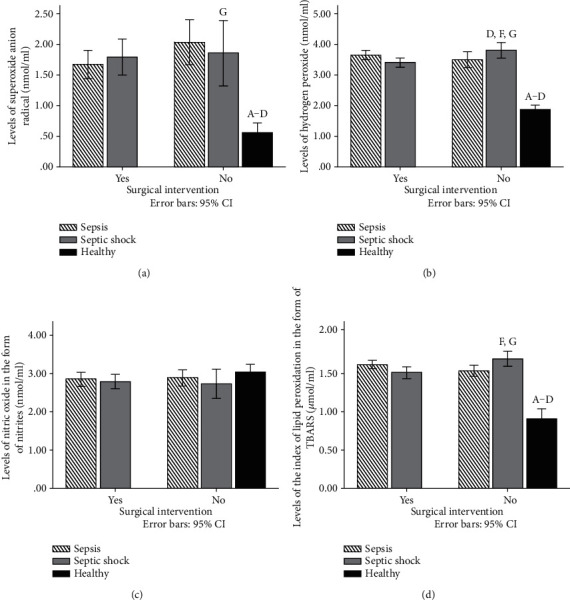
(a) Levels of superoxide anion radical (nmol/ml) in plasma samples of healthy participants and patients with sepsis and septic shock 72 h after admission. (b) Levels of hydrogen peroxide (nmol/ml) in plasma samples of healthy participants and patients with sepsis and septic shock 72 h after admission. (c) Levels of nitric oxide in the form of nitrites (nmol/ml) in plasma samples of healthy participants and patients with sepsis and septic shock 72 h after admission. (d) Levels of the index of lipid peroxidation in the form of TBARS (*μ*mol/ml) in plasma samples of healthy participants and patients with sepsis and septic shock 72 h after admission. Results are presented as mean ± SEM. Statistical significance is considered if the *p* was equal or below 0.05: (A) healthy vs. sepsis operated, (B) heathy vs. sepsis nonoperated, (C) healthy vs. shock operated, (D) healthy vs. shock nonoperated, (E) sepsis operated vs. sepsis nonoperated, (F) shock operated vs. shock nonoperated, (G) sepsis nonoperated vs. shock nonoperated, and (H) sepsis operated vs. shock operated.

Levels of superoxide anion radical were significantly affected in the nonoperated group of patients. Closely, levels of O_2_^−^ were significantly lower in the group of patients with septic shock compared with the sepsis nonoperated group (Figure 1(a)). In the operated group, levels of this marker were not significantly changed. Compared with the healthy participants, the level of this reactive oxygen species was significantly lower than that in other groups.

In comparison to the control group, levels of hydrogen peroxide were significantly higher in all other groups (Figure 1(b)). Also, levels of hydrogen peroxide were significantly increased in the nonoperated group of patients with septic shock compared with the operated group with septic shock and the nonoperated group with sepsis (Figure 1(b)).

On the other hand, bioavailability of nitric oxide was not significantly different between groups (Figure 1(c)).

An index of lipid peroxidation measured in the form of TBARS was measured in all groups, and we have found that the levels of TBARS were significantly higher in the nonoperated group of patients with septic shock compared to the nonoperated sepsis and operated septic shock groups (Figure 1(d)). Also, in comparison with the control group, TBARS was significantly higher in the group of patients with sepsis and septic shock (Figure 1(d)).

### 3.3. Antioxidative Enzyme Activity in Critically Ill Operated and Nonoperated Patients

**Figure 2:**
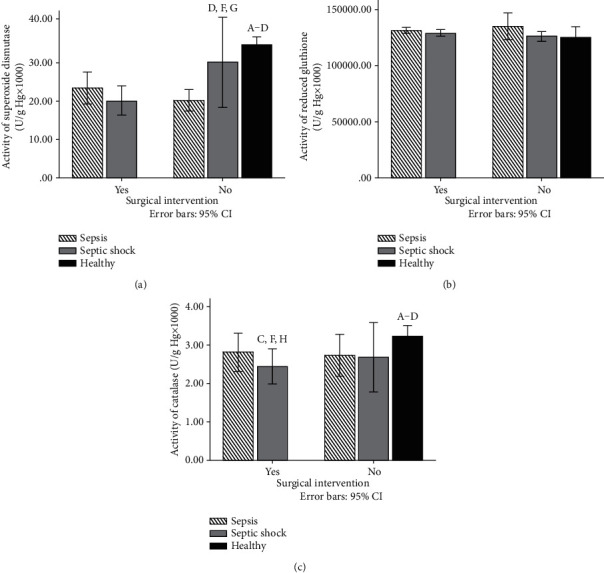
(a) Activity of superoxide dismutase (U/g Hg × 1000) in lysate samples of healthy participants and patients with sepsis and septic shock 72 h after admission. (b) Activity of reduced glutathione (U/g Hg × 1000) in lysate samples of healthy participants and patients with sepsis and septic shock 72 h after admission. (c) Activity of catalase (U/g Hg × 1000) in lysate samples of healthy participants and patients with sepsis and septic shock 72 h after admission. Results are presented as mean ± SEM. Statistical significance is considered if the *p* was equal or below 0.05: (A) healthy vs. sepsis operated, (B) heathy vs. sepsis nonoperated, (C) healthy vs. shock operated, (D) healthy vs. shock nonoperated, (E) sepsis operated vs. sepsis nonoperated, (F) shock operated vs. shock nonoperated, (G) sepsis nonoperated vs. shock nonoperated, and (H) sepsis operated vs. shock operated.

Superoxide dismutase activity was significantly higher in the nonoperated group of patients with septic shock compared to the nonoperated sepsis and operated sepsis groups (Figure 2(a)). Also, catalase activity was changed, and we have found that the lowest levels of this enzyme were in the operated group of patients with septic shock compared with other groups (Figure 2(c)). On the contrary, content of reduced glutathione was not significantly different in our study (Figure 2(b)). Compared with control conditions, we have found that the activity of SOD and CAT was significantly higher in healthy participants than in the group with disease (sepsis or septic shock), while the content of GSH was not significantly different between these groups (Figures 2(a)–2(c)).

### 3.4. PCT and CRP Concentrations in Critically Ill Operated and Nonoperated Patients

**Figure 3:**
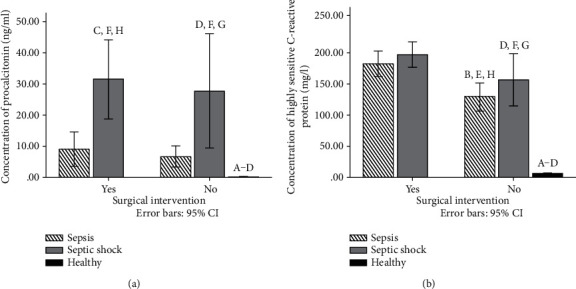
(a) Concentration of procalcitonin (ng/ml) in serum samples of healthy participants and patients with sepsis and septic shock 72 h after admission. (b) Concentration of highly sensitive C-reactive protein (mg/l) in serum samples of patients with sepsis and septic shock 72 h after admission. Results are presented as mean ± SEM. Statistical significance is considered if the *p* was equal or below 0.05: (A) healthy vs. sepsis operated, (B) heathy vs. sepsis nonoperated, (C) healthy vs. shock operated, (D) healthy vs. shock nonoperated, (E) sepsis operated vs. sepsis nonoperated, (F) shock operated vs. shock nonoperated, (G) sepsis nonoperated vs. shock nonoperated, and (H) sepsis operated vs. shock operated.

Procalcitonin was significantly different during septic shock compared to sepsis. Also, in operated and nonoperated groups of patients, patients with septic shock have the highest levels of PCT (Figure 3). In healthy participants, we have confirmed low levels of PCT (below 0.14 ng/ml) at a physiological state (Figure 3).

On the other hand, CRP levels have other dynamics. In nonoperated patients, levels were significantly lower compared with those in operated patients. In these groups, similar concentrations of CRP were observed in patients with sepsis and septic shock (Figure 3(b)). Also, in the control group, we have found low level of hsCRP as we expected (below 6 mg/l) (Figure 3(b)).

### 3.5. Dynamic of the Tested Parameters and Bivariate Correlation Analysis

Table 2 shows differences between sepsis/septic shock and control groups for each parameter in percent. Correlation analysis confirmed that PCT and CRP are in correlation with disease entity (weak, positive). Besides that, PCT in shadow of disease, sepsis or septic shock, was in a positive correlation with superoxide anion radical and TBARS levels. On the other hand, CRP levels were not in association with oxidative stress marker concentrations. During the correlation analysis of activity of antioxidant enzymes with PCT and CRP levels in patients with sepsis and septic shock, we have found that there is no association between these variables in the study population (Tables 3 and 4).

## 4. Discussion

This study was aimed at investigating the relationship between plasma oxidative stress factor levels and organ damage parameters such as PCT and CRP in patients with sepsis and septic shock. Also, this study included operated and nonoperated patients in Intensive Care Units as a therapeutic procedure with unknown prognosis in these critically ill patients.

Sepsis is a potentially life-threatening condition caused by the body's response to an infection. The physiological processes normally neutralize free radicals, and sepsis occurs when the body's response to these high levels of free radicals is out of balance, triggering changes that can damage multiple organ systems [16–18, 20, 28]. Sepsis is the result of an infection and causes drastic changes in the body. It can be very dangerous and potentially life-threatening. Today, we have identified three stages of sepsis: (a) sepsis is when the infection reaches the bloodstream and causes inflammation in the body; (b) severe sepsis is when the infection is severe enough to affect the function of your organs, such as the heart, brain, and kidneys; and (c) septic shock is when a patient experienced a significant drop in blood pressure that can lead to respiratory or heart failure, stroke, failure of other organs, and death [16–18, 20, 28].

On the other hand, oxidants are involved in the formation of deoxyribonucleotides, prostaglandin production, oxidation, and carboxylation and hydroxylation reactions that are essential for normal cell function. Free radicals also participate in the host defense against bacterial infections [28], the regulation of vascular tone, and cell adhesion reactions and act as sensors for oxygen concentration. Reactive oxygen species (ROS) play both physiological and pathophysiological roles in the body. In clinical practice, oxidative stress and its counterpart, antioxidant capacity, can be measured and can guide remedial therapy. Oxidative stress can make a negative impact in all forms of major surgery including cardiac surgery, general surgery, trauma surgery, orthopedic surgery, and plastic surgery. Many and various therapies aimed at reducing oxidative stress in surgery have been tried with variable results. Actually, in surgical patients, the assessment of oxidative stress, improving understanding of its role, both positive and negative, and devising appropriate therapies are of great clinical importance and represent fruitful fields for further research. On the other hand, in different stages of septic disorders, oxidative stress could be different also, and undergoing a surgical intervention could be a tool for reducing oxidative stress in these critically ill patients [10].

Despite the increasing evidence that oxidative stress is a cornerstone on sepsis pathogenesis, the role of oxidative stress in sepsis may be underestimated. For example, in recent sepsis guidelines, its significance has not been highlighted. In this respect, clinicians may not be aware of the potentially pivotal role of oxidative stress in sepsis evolution.

In our study, levels of superoxide anion radical were significantly affected in the nonoperated group of patients. Closely, levels of O_2_^−^ were significantly lower in the group of patients with septic shock compared with the sepsis nonoperated group (Figure 3)). In the operated group, levels of this marker were not significantly changed. Levels of hydrogen peroxide were significantly increased in the nonoperated group of patients with septic shock compared with the operated group with septic shock. In other groups, levels of hydrogen peroxide were not significantly altered (Figure 1(b)).

On the other hand, bioavailability of nitric oxide was not significantly different between groups (Figure 1(c)). The index of lipid peroxidation measured in the form of TBARS was measured in all groups, and we have found that the levels of TBARS were significantly higher in the nonoperated group of patients with septic shock compared to the nonoperated sepsis and operated sepsis groups (Figure 1(d)).

Definitely, levels of prooxidants were higher in the sepsis shock group compared with the sepsis group, as we expected. Oxidative stress mechanisms in sepsis are highly complicated. ROS and RNS play a pivotal role in sepsis evolution, but their specific role and importance remain obscure. The clinical significance of oxidative stress in sepsis is demonstrated by several studies. Cowley et al. found that sepsis survivors had greater antioxidant potential than nonsurvivors and also that it was rapidly raised to normal or supranormal levels [29]. Harmful mechanisms of increased oxidant level in sepsis include modification of proteins, lipids, and nucleic acids contributing to endothelial dysfunction. In addition, the impairment of glycocalyx and the cellular junctions between endothelial cells lead to increased vascular permeability, a cornerstone of sepsis development [30].

Furthermore, the superoxide dismutase activity was significantly higher in the nonoperated group of patients with septic shock compared to the nonoperated sepsis and operated sepsis groups (Figure 2(a)). On the contrary, content of reduced glutathione was not significantly different in our study (Figure 2(b)). Also, catalase activity was changed, and we have found that the lowest levels of this enzyme were in the operated group of patients with septic shock compared with other groups (Figure 2(c)). In a previous study, the prognostic potential of the antioxidant enzymes superoxide dismutase (SOD) and catalase (CAT) was evaluated in sepsis [31]. Enzyme concentrations were determined in samples obtained from septic patients at the time of diagnosis. Statistically significant increases in activities of total plasma SOD and CAT were found in septic patients when compared with healthy adult controls [31]. Similar to our results, sepsis and septic shock were associated with lower levels of these enzymes with respect to healthy participants. Also, the surgical intervention was the main factor for changing the levels of prooxidants. Most importantly, during severe forms of sepsis, we have found different activities of antioxidant enzymes. Also, it is difficult to say which degree is changed because of the absence of an adequate control (healthy) group of patients, but there is some evidence regarding the disturbance of redox status in critically ill patients. Other authors confirmed our results [32]. The superoxide dismutase activity catalyzes the dismutation of O_2_ to H_2_O_2_, CAT mediates the breakdown of H_2_O_2_ to water (H_2_O) and oxygen, and GPx helps in the conversion of H_2_O_2_ into H_2_O. There is a balance between generation of oxidants and their clearance by antioxidants under normal conditions. Definitely, in patients with sepsis shock, we have observed imbalanced redox homeostasis and thus poorer prognosis of disease.

Procalcitonin (PCT) is a biomarker that exhibits greater specificity than other proinflammatory markers (e.g., cytokines) in identifying sepsis and can be used in the diagnosis of bacterial infections [33]. Procalcitonin is also produced by the neuroendocrine cells of the lung and intestine and is released as an acute-phase reactant in response to inflammatory stimuli, especially those of bacterial origin. This raised procalcitonin level during inflammation is associated with bacterial endotoxin and inflammatory cytokines. In our study, correlation analysis confirmed the connection between PCT and oxidative stress markers [33]. As in a previous study, the process of lipid peroxidation seems to correlate with the degree of infection as indicated by PCT levels [34]. Because of the high mortality rate in patients with sepsis and septic shock, it is very important information that PCT is a good prognostic marker. As we found, procalcitonin could be a predictor of the disease course, as well as prognosis in different stages of septic disorders. Recent studies have shown a significant correlation of procalcitonin (PCT) with infection and suggest that PCT is useful for the early diagnosis of systemic infection [35]. Early diagnosis and prompt treatment for infection will significantly improve the prognosis and avoid the unnecessary use of antibiotics for patients without infections.

On the other hand, CRP was not sensitive to contribute to the course of the disease in these patients. CRP sensitivity must be further evaluated by selecting cut-off values in various infections. Still, CRP has mild sensitivity but just in acute infection without specificity, with no focus on the targeted organ's damage. Because of those facts, CRP is likely to be outside of a clinical setting in critically ill patients.

## 5. Conclusion

PCT has confirmed a positive correlation with prooxidants and type of critical illness, and performing surgical intervention diminished oxidative stress in patients with septic shock. Prognosis in critically ill patients was strongly associated with PCT levels but not with nonspecifically C-reactive protein.

## Figures and Tables

**Table 1:** Distribution of gender and age in the study population. Gender is presented as frequency in percent (%), and age is presented as mean ± standard deviation in years. For testing the statistical differences between groups, we used the chi-squared test (gender) and Kruskall-Wallis nonparametric test (age).

Group/parameter	Healthy (*n* = 17)	Sepsis operated (*n* = 24)	Septic shock operated (*n* = 25)	Sepsis nonoperated (*n* = 26)	Septic shock nonoperated (*n* = 28)
Gender (m/f)	m 10 (58.8%)	m 11 (45.8%)	m 13 (52.0%)	m 14 (53.8%)	m 15 (53.6%)
	f 7 (41.2%)	f 13 (54.2%)	f 12 (48.0%)	f 12 (46.2%)	f 13 (46.4%)
Age (years)	56.02 ± 1.02	62.02 ± 2.34	59.32 ± 1.98	65.87 ± 3.32	68.05 ± 3.55

**Table 2:** Dynamics of all the tested parameters in the group of patients with sepsis or septic shock in percent (%) compared with the control group (healthy participants).

Parameter	Group	Mean	Std. deviation	Std. error	Percent (%)
Superoxide anion radical	Sepsis	1.82	1.38	0.10	+231.7%
	Septic shock	1.80	1.36	0.13	+226.9%
	Healthy	0.55	0.15	0.06	0.0%

Hydrogen peroxide	Sepsis	3.58	0.96	0.07	+91.5%
	Septic shock	3.49	0.72	0.07	+86.7%
	Healthy	1.87	0.14	0.06	0.0%

Nitrites	Sepsis	2.76	0.90	0.07	-5.8%
	Septic shock	2.68	0.86	0.08	-8.7%
	Healthy	2.94	0.18	0.07	0.0%

Index of lipid peroxidation	Sepsis	1.52	0.28	0.02	+76.4%
	Septic shock	1.49	0.34	0.03	+73.3%
	Healthy	0.86	0.13	0.05	0.0%

Superoxide dismutase	Sepsis	20.90	16.80	1.28	-37.0%
	Septic shock	21.42	20.55	1.93	-35.4%
	Healthy	33.17	1.82	0.74	0.0%

Reduced glutathione	Sepsis	132181.85	35464.72	2712.05	+6.3%
	Septic shock	127675.74	12944.06	1212.32	+2.7%
	Healthy	124313.54	9297.29	3795.60	0.0%

Catalase	Sepsis	2.76	2.44	0.19	-14.1%
	Septic shock	2.49	2.18	0.20	-22.6%
	Healthy	3.22	0.28	0.11	0.0%

Procalcitonin	Sepsis	7.97	22.95	1.70	+5952.2%
	Septic shock	30.46	57.09	5.32	+23033.9%
	Healthy	0.13	0.01	0.01	0.0%

C-reactive protein	Sepsis	151.86	105.83	7.60	+2556.5%
	Septic shock	178.97	101.85	9.18	+3030.7%
	Healthy	5.72	0.77	0.31	0.0%

**Table 3:** Bivariate (Pearson) correlation analysis between oxidative stress markers, PCT, CRP, and stage of disease (sepsis or septic shock). Correlation is considered present if the *p* value was equal or below 0.05 (*p*), and the degree and direction of association were expressed as the coefficient of correlation (*R* values always range between -1 (strong negative relationship) and +1 (strong positive relationship), and *R* values at or close to zero imply weak or no linear relationship).

Variable		Disease	O_2_^−^	H_2_O_2_	NO	TBARS	PCT	CRP
Disease	*R* *p*	/	0.009 0.874	0.050 0.394	0.048 0.418	0.043 0.471	0.266 0.000^∗∗^	0.126 0.025^∗^
O_2_^−^	*R* *p*		/	0.047 0.428	0.082 0.163	0.050 0.397	0.010 0.289	0.080 0.172
H_2_O_2_	*R* *p*			/	0.228 0.000^∗∗^	0.396 0.000^∗∗^	0.018 0.759	0.052 0.376
NO	*R* *p*				/	0.443 0.000^∗∗^	0.039 0.512	0.003 0.964
TBARS	*R* *p*					/	0.133 0.024^∗^	0.053 0.366
PCT	*R* *p*						/	0.247 0.000^∗∗^
CRP	*R* *p*							/

**Table 4:** Bivariate (Pearson) correlation analysis between antioxidant enzymes, PCT, CRP, and disease entity. Correlation is considered present if the *p* value was equal or below 0.05 (*p*), and the degree and direction of association were expressed as the coefficient of correlation (*R* values always range between -1 (strong negative relationship) and +1 (strong positive relationship), and *R* values at or close to zero imply weak or no linear relationship).

Variable		Disease	SOD	GSH	CAT	PCT	CRP
Disease	*R* *p*	/	0.014 0.814	0.077 0.195	0.057 0.337	0.266 0.000^∗∗^	0.126 0.025^∗^
SOD	*R* *p*		/	0.007 0.909	0.001 0.998	0.027 0.652	0.001 0.297
GSH	*R* *p*			/	0.048 0.421	0.052 0.386	0.053 0.374
CAT	*R* *p*				/	0.023 0.696	0.007 0.906
PCT	*R* *p*					/	0.247 0.000^∗∗^
CRP	*R* *p*						/

## Data Availability

All data are available.
